# Associating COVID-19 Severity with Urban Factors: A Case Study of Wuhan

**DOI:** 10.3390/ijerph17186712

**Published:** 2020-09-15

**Authors:** Xin Li, Lin Zhou, Tao Jia, Ran Peng, Xiongwu Fu, Yuliang Zou

**Affiliations:** 1 School of Architecture and Civil Engineering, Xiamen University, Xiamen 361005, China; lixin99182@xmu.edu.cn; 2 School of Urban Design, Wuhan University, Wuhan 430072, China; 2014301530136@whu.edu.cn; 3 School of Remote Sensing and Information Engineering, Wuhan University, Wuhan 430072, China; 4 School of Civil Engineering and Architecture, Wuhan Institute of Technology, Wuhan 430074, China; 15050101@wit.edu.cn; 5 Information Department, Wuhan Land Use and Urban Spatial Planning Research Center, Wuhan 430010, China; fuxiongwu@wlsp.org.cn; 6 School of Health Sciences, Wuhan University, Wuhan 430071, China; zouyl@whu.edu.cn

**Keywords:** COVID-19, Weibo data, epidemic analysis, urban spatial patterns, Wuhan

## Abstract

Wuhan encountered a serious attack in the first round of the coronavirus disease 2019 (COVID-19) pandemic, which has resulted in a public health social impact, including public mental health. Based on the Weibo help data, we inferred the spatial distribution pattern of the epidemic situation and its impacts. Seven urban factors, i.e., urban growth, general hospital, commercial facilities, subway station, land-use mixture, aging ratio, and road density, were selected for validation with the ordinary linear model, in which the former six factors presented a globally significant association with epidemic severity. Then, the geographically weighted regression model (GWR) was adopted to identify their unevenly distributed effects in the urban space. Among the six factors, the distribution and density of major hospitals exerted significant effects on epidemic situation. Commercial facilities appear to be the most prevalently distributed significant factor on epidemic situation over the city. Urban growth, in particular the newly developed residential quarters with high-rise buildings around the waterfront area of Hanyang and Wuchang, face greater risk of the distribution. The influence of subway stations concentrates at the adjacency place where the three towns meet and some near-terminal locations. The aging ratio of the community dominantly affects the hinterland of Hankou to a broader extent than other areas in the city. Upon discovering the result, a series of managerial implications that coordinate various urban factors were proposed. This research may contribute toward developing specific planning and design responses for different areas in the city based on a better understanding of the occurrence, transmission, and diffusion of the COVID-19 epidemic in the metropolitan area.

## 1. Introduction

In the new era of the 21st century, despite great progress in medical technology, vaccination programs, and public health systems [1], severely infectious diseases, such as the severe acute respiratory syndrome (SARS-CoV 2002), the novel swine-origin influenza-A virus (H1N1 2009), and the middle east respiratory syndrome (MERS-CoV 2012), have never disappeared but evolved into novel ones and caused a series of public health consequences, which have demonstrated that infectious events could develop rapidly to interfere with the social order and even threaten global stability [2,3,4].

By 18 August 2020, the novel severe acute respiratory syndrome coronavirus (SARS-CoV-2), i.e., the coronavirus disease 2019 (COVID-19), had developed into a global pandemic, causing over 22 million confirmed cases and 0.78 million deaths worldwide. The impact of the COVID-19 pandemic is the most significant flu pandemic since 1918, resulting in a public health social impact, including public mental health. Wuhan, the capital city of Hubei province in China, was under siege when encountering the first round of sudden attack from COVID-19, which almost pushed the local medical system to the brink of collapse [5]. The number of confirmed cases in Wuhan accounted for over 60% of all cases in China by the end of March, 2020. 

Compared to the last century, although our modern society has become more robust and resilient to deal with unexpected hazards, extra attention should be paid to urban factors and the built environment for the purpose of public health and security. Meanwhile, urban expansion and population agglomeration have increased the risk of encountering epidemic diseases in urban settings, as over half of the world’s population (55%) had moved to cities by 2018 and this proportion is anticipated to reach to 68% by 2050 [6]. The ongoing pandemic of COVID-19 reminds us of the strong impact of massive urbanization, which has changed traditional society. Imperative updates to urban systems are needed to prevent damage from severe infectious diseases. In order to reduce the threat that infectious diseases pose to the metropolitan area with a large population, specific planning strategies adapted to urban settings should be considered based on a better understanding of how infectious diseases transmit in urban environments [1]. 

In the first two months since the outbreak of COVID-19, a plethora of epidemiological investigations were made, mainly focusing on the disease characteristics, pathogenic mechanism, and population distribution [6,7,8,9]. As reported, COVID-19 is an enveloped non-segmented positive-sense RNA virus that belongs to the family Coronaviridae and the order Nidovirales [10]. This novel coronavirus can cause lower respiratory tract disease with poor clinical outcomes. At the onset of infection, clinical studies of hospitalized patients frequently show symptoms associated with viral pneumonia, most commonly fever, cough, sore throat, and fatigue [11]. Although nucleic acid testing is required for the confirmation of COVID-19 infection, clinical diagnosis could also be made based on a comprehensive judgment of symptoms, exposures, and chest imaging. To date, supportive care for patients is typically the standard protocol because no specific effective antiviral therapies have been identified. Although most human coronavirus infections are mild, the epidemics of the several betacoronaviruses, such as SARS-CoV, H1N1, and MERS-CoV, could cause high mortality. The current COVID-19 is caused by SARS-CoV-2, which is similar to the above ones, but some noticeable differences also exist [12]. Previous experience with controlling coronavirus diseases has proved that effective prevention in advance is more constructive, before too many people have been affected. Therefore, we face an urgent demand to investigate the spatial pattern of epidemiology and its potential impact on urban lives. Characterizing human–space interactions within urban settings contributes to the prevention of disease transmission (not only with regard to COVID-19, but also other infectious diseases) by actively navigating human-mediated processes [13,14]. Analyzing environmental factors is an indispensable component of understanding the mechanism of transmission of the infectious disease [15].

As researchers found out, COVID-19 spreads faster and more easily than its ancestor SARS-CoV 2002, mainly through respiratory droplets of pathogen carriers [7]. In confined indoor spaces or shared environments with crowded people, potential aerosol transmission of COVID-19 could be possible, almost catching up to the spread rate of common influenza. The idea of spatial diffusion, i.e., an outward movement from a single spot to a broader coverage, could be used to elucidate how the disease spreads in the city through urban flows. Different from the ideal model of even exposure to infectious agents within a homogenous environment, variations in potential disease transmission always happen due to various urban factors [16,17], resulting in highly heterogeneous infection patterns within the city [18,19].

Previous studies on SARS-CoV 2002 have provided evidence to establish associations between the epidemic incidence and urban factors, inferring that the spread of COVID-19 may also have similar multidimensional characteristics, which could be reflected in demographic conditions, proximity, transportation, and socioeconomic activities [20]. For instance, the experience of Toronto medical systems that fought against SARS-CoV 2002 indicated that hospitals were the major source that introduced higher risks to their surroundings, as the contaminated environment not only facilitates virus transmission among health care workers, patients, and hospital visitors, but also make more people and families exposed to the risk if pathogen carriers move around the hospital [21]. The clinical uncertainty of such a novel virus makes it more difficult to seize the window period of early control at the beginning of the outbreak. Some other spatial-related dimensions in metropolitan areas could also be recognized as essential factors to evaluate the transmission mechanism of COVID-19 in the urban context, as the density of residential blocks, shopping amenities, and the ration relying on mass transportations is extremely high, creating a suitable urban environment for the disease transmission [20]. Moreover, along with the influx of urban migrants, the squatter settslements with limited housing conditions, sanitation facilities, and access to urban infrastructures, may change the pattern of risks [6]. 

In previous studies, the investigations on the spatiotemporal nature of coronaviruses mainly focused on the provincial or national level [22,23], whereas few studies have been made to address the impacts on the city level, particularly based on patients’ living locations. Identifying the impacts of urban environments becomes increasingly important to control risks in the current circumstance. However, it is difficult to evaluate how these impacts vary on the city level because previous epidemiology investigations used to focus on a coarse resolution across provincial or regional boundaries. Therefore, a more incisive investigation with credible location-based details is imperative to further explore the distribution pattern of COVID-19 and influencing urban factors on the city level. We aim to extend the understanding of this disease and its spatial impact not only by validating important urban factors, but also by providing convincing evidence to identify corresponding locations where these factors shed significant influences. Instead of providing a universal antidote, a more precise strategy could be proposed for different areas to better deal with similar public health emergencies in the future, if the spatial variation is taken into consideration.

## 2. Materials and Methods 

### 2.1. Study Area

**Figure 1:**
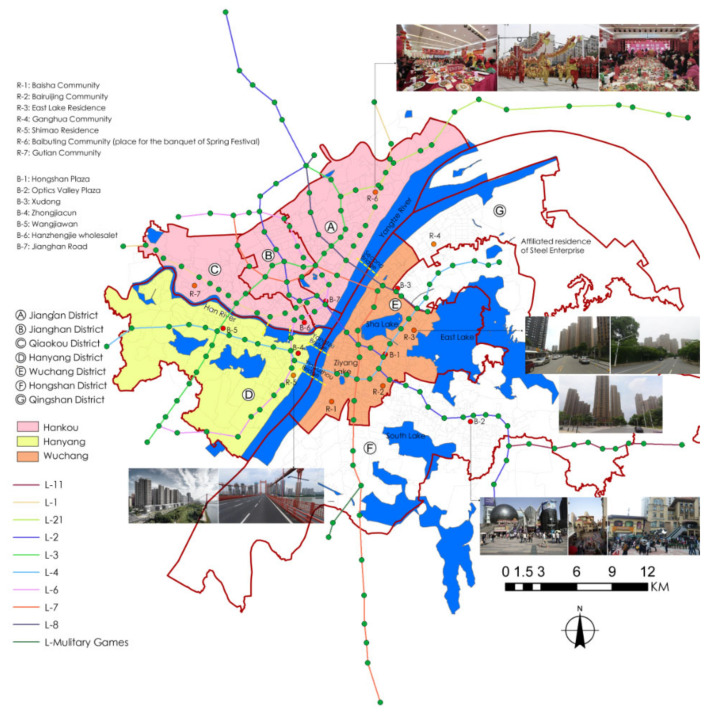
The basic geographic information of the study area and characteristics of urban factors.

Wuhan, the capital city of Hubei Province in central China, lies at the confluence of the Yangtze River, the longest in China, and its longest tributary, the Hanjiang River. These two great waterways split the metropolitan areas into three parts, namely Wuchang, Hankou, and Hanyang (Figure 1). It is also home to China’s largest urban lake: East Lake. With the water taking up one fourth of the total urban area, Wuhan is hailed as a city of lakes. We select the third-ring core area within Wuhan as the study area, covering seven administrative districts with an area of about 860 square kilometers. 

In Wuhan, the north region is different from the south in terms of urban pattern, spatial form, socio-economic situation, etc. The north bank contains Hanyang and Hankou, and the later consists of three administrative Districts, i.e., Jiang’an, Jianghan, and Qiaokou. As a well-developed commercial centre, Hankou occupies a flat terrain with few natural obstacles but convenient transportation infrastructures. In particular, highly concentrated populations and development intensity can be found at the old downtown area of Hankou, with an agglomeration of urban facilities, such as shopping malls, general hospitals, and subway stations. This area also features a high aging ratio.

Situated at the west part of the city, Hanyang initially operated as an industrial zone in the early 19th century. After the completion of the first bridge on the Yangtze River in the 1950s, Hanyang became the hinge place to connect the other two towns. Due to the adequate stock of reserved land and preferential policy support, the riverside area of Hanyang has kept upgrading along with the prosperous real estate market in recent years, particularly for newly developed residential projects of high-rise buildings. 

On the south bank are three administrative Districts, among which Wuchang is part of the birthplace of Wuhan city. Between the Sha Lake and the Ziyang Lake is another old downtown area, which also possesses highly concentrated urban facilities and aging ratio, whereas the intensity is slightly lower than that of the Hankou downtown area. The strip zone between the riverside area and the East Lake is another important place for newly developed real estate projects. As regards the other two Districts, Hongshan was the outer city of Wuchang, but in recent years it has gradually evolved into a sub-centre where the city extends to the east, and Qingshan is based on the compound of the large state-owned enterprise, Wuhan Steel Co., and its affiliated residential area. 

### 2.2. Data Source

At the beginning of February 2020, medical recourses were seriously squeezed within a short period of time and the hospital reached its capacity to treat patients. Many suspected or confirmed patients with mild to moderate syndrome had to stay at home. Some of them might have had to go outside for different reasons, facilitating the spread of the disease. More seriously, some patients’ situation was exacerbated because staying at home cannot be rigorously enforced with effective health care and treatment. Under such a situation, Sina Weibo opened an online channel for people to seek help. To ensure the authenticity of help-seeking information, it was necessary to provide the help-seeker’s personal information, including name, age, gender, dwelling address, contact information, syndrome description, etc. Some designated departments were involved to check the authenticity of the information before it was posted online. Therefore, the Weibo help data can be used to estimate the severity of the epidemic in Wuhan with precise positions. Until the middle of February 2020, when many shelter hospitals were built to admit these patients, Weibo was one of the most important channels for help-seeking.

**Figure 2:**
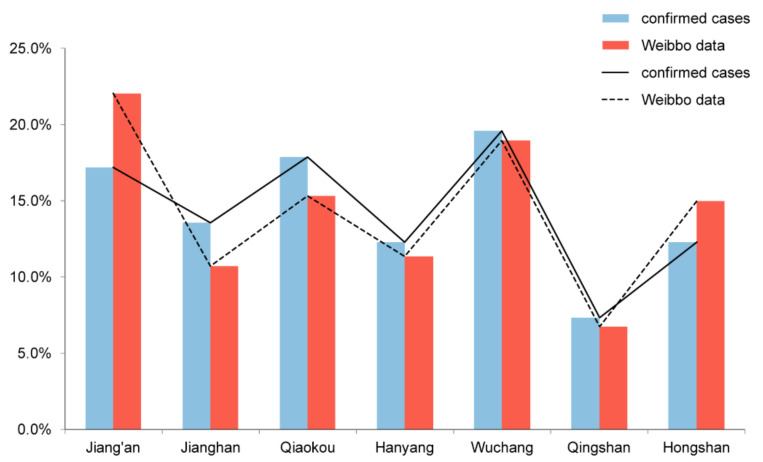
Comparison between the Weibo data and the confirmed cases.

We collected the open data from Weibo help channel from 23 January to 13 February 2020, as it was the most severe period of the epidemic in Wuhan. The patient’s address was converted to the World Geodetic System 1984 (WGS-84). Eventually, a total number of 327 locations (families) within the Wuhan third-ring area were identified. The information of a total of 415 infected persons was collected, with an average age of 59.9 years old. To ensure the representativeness of sampling, we compared the distribution of Weibo help data with that of the confirmed cases according to the jurisdiction, and it shows strong consistency between the two datasets (Table 1, Figure 2). The Chi-square test further confirmed that there was a non-significant difference (Chi-square = 9.977, *p* = 0.126). In addition, other basic spatial data were collected, such as digital maps, the shapefile of the features within the study area, point of interest (POI), and population data on the community level.

### 2.3. Outcome Variable

As the study area covers 1025 urban communities, the Weibo help data appear sparsely distributed in the urban space. Unexpected bias might exist if the community is adopted as the statistical unit. In addition, these communities are subject to significant variation in size and shape, and it is difficult to conform with the statistical requirements. Therefore, we decided to divide the study area into uniform grids according to Griffith’s method [24]. After removing some grids with substantial vacant land and water, 502 urban grids of 1 km^2^ coverage were eventually adopted for statistical analysis. The kernel density of Weibo help data was calculated to denote the epidemic severity, which denotes the degree of impact of COVID-19 in a given spatial unit. When adopting the Weibo help data, the epidemic severity refers to the number of potential infected population and social panic, as this means people have to try informal ways to seek help in such an emergent situation. This method is beneficial not only for eliminating a large number of invalid statistical units, but also for presenting the pattern of epidemic spread in the city with good spatial continuity.

Further, we also adopted kernel density as the variable to associate epidemic severity with urban factors. According to the Tobler’s First Law of Geography [25], for any given incidence, there is a spatial probability around the place of occurrence. Conceptually, we can fit a smoothly curved surface over this given point to represent the probability of occurrence, which diminishes along with increasing distance away from this point. As the epidemic is associated with all kinds of social activities which rely on different urban facilities to take place, in this sense, although the location of Weibo data is based on the home address of confirmed or suspected cases, the potential association between the Weibo data and hospitals could be reasonable, because our method is also based on the probability rather than the incidence itself [26].

### 2.4. Urban Factors

Since COVID-19 is a novel coronavirus which is homologous to SARS-CoV 2002, the rationale of adapting the transmission mechanism of SARS-CoV 2002 as highly related influencing factors, i.e., hospitals, vehicles, public places, households, should be reasonable for an explorative investigation [20].

**Figure 3:**
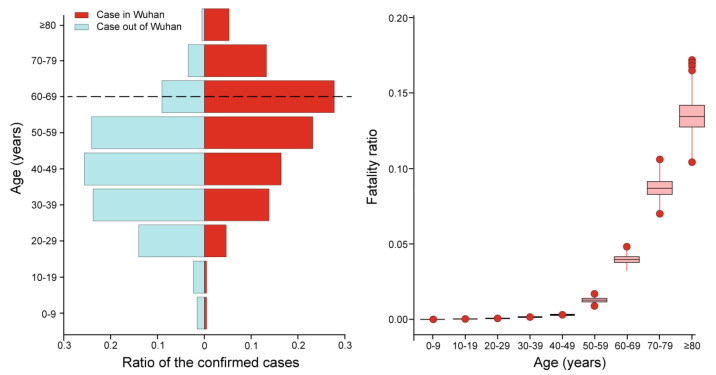
The confirmed cases by age and fatality ratio (adapted from Verity, et al., 2020).

Eventually, seven urban factors among four categories of indicators, socio-demographic, urban growth, urban facilities and land-use, were selected for testing [6]. For instance, infectious diseases are usually associated with the distribution of population density in the urban context. In particular, elderly groups have been subjected to the highest level of risk among the cases in Wuhan (Figure 3) [10]. According to a recent study with a large sample size of 1099 COVID-19 patients, people over 60 years old suffered from the highest risk of severe symptoms and mortality due to COVID-19 infection [9]. Those elder people with underlying conditions, such as hypertension, diabetes, cardiovascular disease, and chronic respiratory disease faced more exposure because of frequent visits to the hospital. Therefore, we took the community aging ratio, i.e., the proportion of elderly people over 65 years old within the community, as the socio-demographic indicator in this study. In this study, the population density was excluded from the model to reduce the multicollinearity problem due to the high correlation with the aging ratio.

With regard to the indicators of urban growth, two factors, i.e., floor area ratio (FAR) and road density, were adopted for modeling. These two factors are also suitable proxy variables of population density without introducing multicollinearity. Indicators of urban facilities included the density of general hospitals, commercial facilities, and subway stations. Among them, hospital density denotes the risk of contamination during medical services or due to adjacencies, and the other two factors denote the major public contact with people in urban environments. Besides these, the degree of land-use mixture was calculated based on the Shannon Entropy to denote the diversity of urban functions. All these urban factors are listed in Table 2.

As no serious multicollinearity was found among the seven urban factors, they were all included in the model for testing at the beginning. The significance of variables and the values of variance inflation factor (VIF), which is an indicator that measures the degree of multicollinearity, were analyzed to screen out invalid factors.

### 2.5. Method

In the analysis process, the Weibo help data were geo-coded with WGS-84 and visualized on the map in the ArcGIS Desktop software (version 10.3, Environmental Systems Research Institute, Inc., Redlands, CA, USA). The kernel density analysis was conducted with the geo-coded data before statistical models were established.

The spread of infectious epidemic is related not only to human interaction, but also to the urban environments in which social activities took place. Through establishing models, we can verify the impacts of various urban factors on the distribution of disease. Therefore, the overall effects of urban factors could be tested with the ordinary least squares (OLS) coefficient estimates, assuming that the effects are evenly distributed in urban space. However, the factor of population density itself may not necessarily result in a serious epidemic situation. On this basis, by calculating Moran’s I, we can determine whether and to what extent the epidemic severity and these urban factors are subject to spatial autocorrelation. 

If Moran’s I is significant, meaning that the OLS model with global estimation cannot fully explain the spatial heterogeneity of influencing factors, then we will adopt the geographically weighted regression (GWR) for local estimation. In the GWR model, we assume that the regression coefficient is a spatial function of the observation point (focal point). In other words, the regression coefficients are no longer fixed as the constants of the global estimation, but change by adopting adjacent neighbors of the designated focal point as sub-samples in the local regression. As the GWR model incorporates the spatial characteristics of independent variables for the local estimation, it could be used to detect the heterogeneity of disease severity along with the environment variation in different regions. The function of GWR is as follows

Yi = β0 (μi, νi) + Σ βj (μi, νi) Xij + εi(1)

where Yi denotes the outcome variable of the spatial unit i; Xij denotes the urban factor in this study; βj (μi, νi) is the variable estimate, and εi is the random error term. The estimated value of βj changes with the spatial weight matrix W(μi, νi), in which the attenuation function f (d) could be defined as

f (d) = (1 − d2/h2)2, d < h or f (d) = 0, d ≧ h(2)

where d is the distance between unit i and j; and h is the bandwidth which could be determined by using the Akaike Information Criterion (AIC). When the W(μi, νi) is obtained, βj (μi, νi) could be estimated by using the standard weighted least square regression with the following function

βj (μi, νi) = [XT W(μi, νi) X] − 1 XT W(μi, νi) Y(3)

## 3. Results

### 3.1. Distribution of Cases and Urban Factors

**Figure 4:**
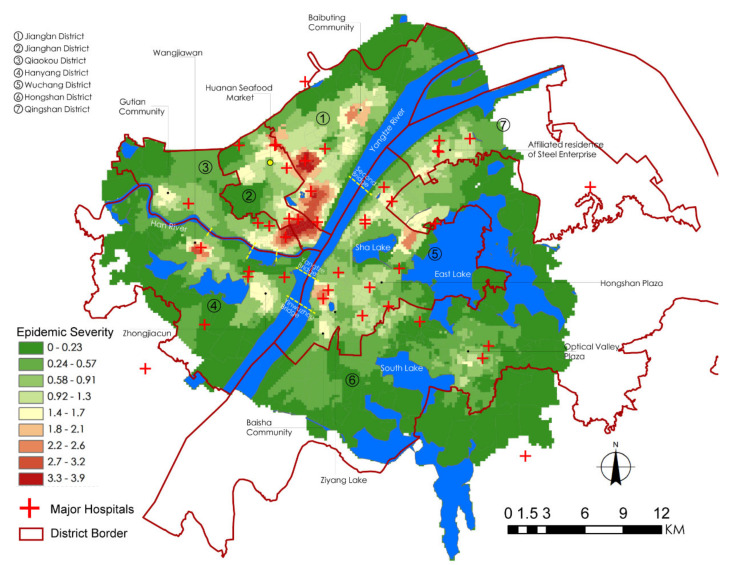
Kernel density of the epidemic severity based on Weibo help data.

**Figure 5:**
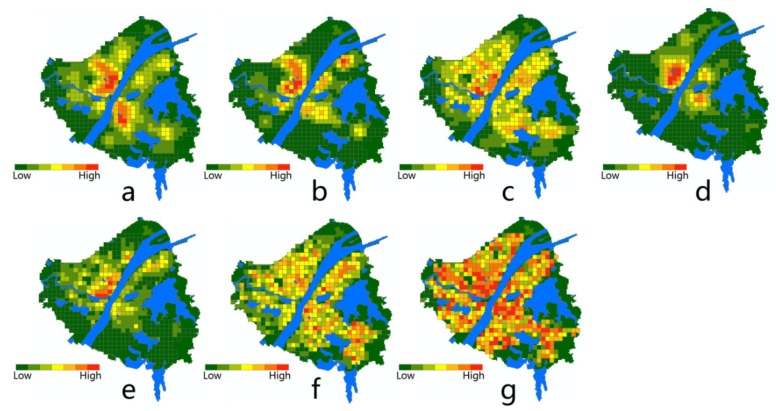
Kernel density of the selected urban factors, (**a**) commercial; (**b**) hospital; (**c**) FAR; (**d**) subway station; (**e**) community aging ratio; (**f**) road density; (**g**) land-use mixture.

Before modeling the association between the epidemic severity and urban factors, it is helpful to reveal some intuitive information by comparing their spatial patterns. According to the distribution of Weibo help data, we can estimate the severity of COVID-19 in the core area of Wuhan city (Figure 4). The help-seeking data mostly concentrated at Hankou town on the north bank of the Yangtze River, especially at the adjacencies of Jiang’an, Jianghan, and Qiaokou jurisdiction. As found, Hankou is the place where commercial facilities, hospitals, subway stations, and aging communities highly concentrate, whereas FAR, road density, and land-use mixture seem more dispersed in the urban space (Figure 5). The epidemic situation developed from Hua’nan Seafood Market, in which the outbreak was detected, all the way to the south, spreading along the highly concentrated commercial areas such as Jianghan business loop, Jiefang Avenue, and the large surrounding areas of Hanzheng wholesale market. The hotspot pattern of severity is highly consistent with that of the major hospital and the commercial facility (Figure 5). The epidemic situation seemed also widespread in the vast area along the Yangtze River and the Han River. Another spreading trajectory went along the north bank of the Yangtze River (the subway line-1), ending with a hotspot with high incidence around the Baibuting community. A sporadic situation was found at the Gutian community on the upper stream of the Han River. In Hanyang District, the epidemic mainly concentrated around two business centers, i.e., Zhongjiacun and Wangjiawan.

The overall severity of the epidemic on the south bank of the Yangtze River was much lower than that on the north bank. One of the strip areas with concentrated incidence went along the Yangtze River waterfront, extending from the Baisha community at the south to the northern area of Qingshan District, where the Wuhan Steel compound and the affiliated residential areas (Ganghua Community) are located. These two places are also where major hospitals and commercial facilities concentrate. Several hotspots with high incidence were detected mainly around the important nodes that closely connect to Hankou or Hanyang, such as the Renmin Hospital of Wuhan University near the Yangtze Bridge, and Xudong commercial loop near the Second Yangtze Bridge. The epidemic continued to develop until reaching a sub-climax in the surroundings with high residential density between the Sha Lake and the East Lake. Although the epidemic severity attenuated during its spread from Wuchang to the hinterland of Hongshan because of the restriction of natural environments, some hotspots fluctuated around the commercial nodes or densely populated places, such as Hongshan Plaza, Bairuijing Central Community, Optics Valley Plaza.

### 3.2. OLS Model

For the OLS model, the F-test is significant at a 99% confidence level (Table 3), indicating that at least one factor has a significant influence on the epidemic. The model presents a moderately high explanatory power with 64% of the variation explained by the selected factors, among which aging, FAR, and commercial density are highly significant at the 99.9% confidence level (*p* < 0.001). The coefficients of hospitals, subway stations, and land-use mixture are significant at the 99% confidence level. Among them, the coefficients of aging, FAR, commercial density, hospital density, and subway station density, are positive, indicating that these factors were associated with epidemic severity and might incur more help-seeking. Conversely, the coefficient of land-use mixture is negatively associated with the epidemic severity. However, the association between road density and the epidemic severity is non-significant.

In the Null model with only the outcome variable, the Moran’s I index was 0.796 (*p* < 0.001), indicating a significant spatial autocorrelation. For the OLS model, the Moran’s I index was reduced to 0.611 (*p* < 0.001). Therefore, it is still necessary to consider other alternative means to eliminate the estimation error caused by spatial heterogeneity, so as to optimize the model fit.

### 3.3. GWR Model

**Figure 6:**
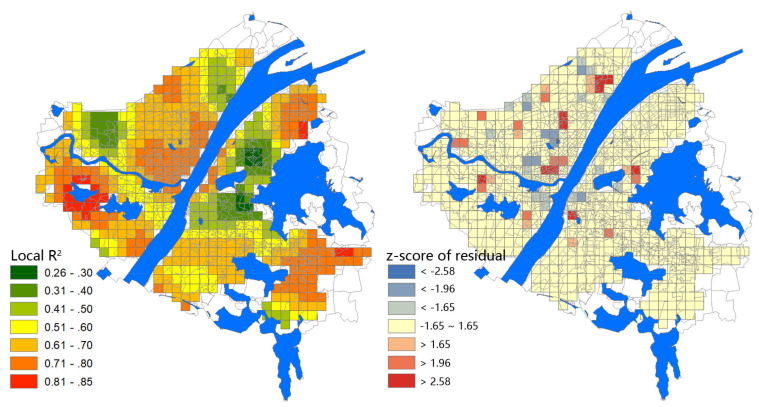
The local R2 value of the geographically weighted regression (GWR) model and the residual standard deviation with z-score.

As the factor of road density presented non-significant impact on the epidemic in the OLS model, this factor was removed from the GWR model to reduce the model complexity. The result shows that the GWR model has an average adjusted R2 of 0.822, indicating a very high explanatory power (Table 4). Through analyzing the significance graph of local R2 (Figure 6), the data in most grids can be well fitted with the GWR model. The model fits were over 0.65 for most of the grids at Hankou, where the epidemic situation was the worst and most concentrated, and the distribution of areas with high local R2 value is highly consistent with the places of high incidence, indicating that the selected urban factors could well explain the epidemic situation in this region. Even for those grids in which the model-fit is very low, the local R2 value is mostly close to 30%, which still maintains an acceptable level. Compared to the OLS model, the adjusted R2 of the GWR model is significantly improved and the residual is effectively reduced.

We can also compare the model-fit by using the Akaike Information Criterion (AIC), which can be applicable to any sample size [27]. A decrease in AICc by more than three points could be regarded as a significant improvement for the model performance [28]. In this study, the AICc of the GWR model is decreased by nearly 257 compared with that of the OLS model, which means the model-fit is improved by about 80%. In addition, the Moran’s I of the GWR model is reduced to 0.314 (*p* < 0.001), which means the spatial autocorrelation is reduced by 48.6% compared with the OLS model (Table 5). Therefore, these results reveal that the GWR model is a better choice.

The regression coefficients of the six factors are subject to some degrees of variation for different grids, and even present opposite effects. The distribution of variable significance also presents noticeable spatial heterogeneity (Figure 6), indicating that these factors have non-stationary influences on the epidemic in different areas.

### 3.4. The Influence of Hospitals

**Figure 7:**
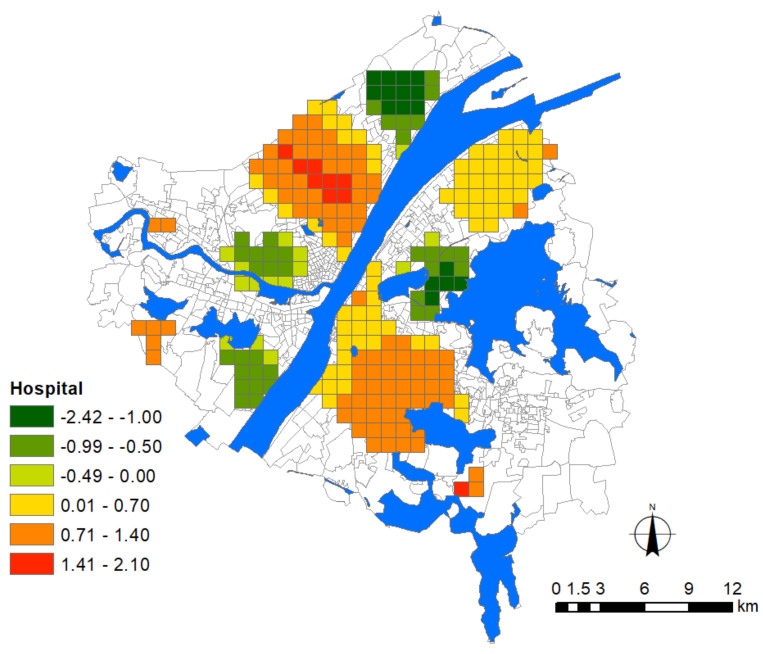
Areas significantly affected by the hospital factor. The legend denotes the corresponding intensity of influence.

By comparing the locations of major hospitals (including the first group of designated fever hospitals) with the epidemic distribution (Figure 4 and Figure 5), we found that these hospitals are all located within or close to the epidemic hotspots (Figure 4 and Figure 5), and formulated the hypothesis that major hospitals probably intensified the spread of COVID-19 to the surrounding areas in the early stage. As people in China prefer general hospitals to have medical services, during the outbreak of COVID-19, which coincided with the flu season, a large number of people with a high frequency of hospital visits may cause extra risk of cross-infection around these places. Such a hypothesis could be supported as the GWR model shows that the influence of hospitals on epidemic severity is indeed unevenly distributed in the city, resulting in three agglomeration areas with statistical significance. Statistical evidence reveals that the epidemic severity is highly associated with the distribution of major hospitals, particularly in Hankou (Figure 7). The spatial pattern is in agreement with the hypothesis that the epidemic risk diffused from the place where the first several confirmed cases were found, i.e., Hua’nan Seafood Market (Figure 4), perpendicularly to the waterfront area of the Yangtze River. The risk is concentrated along with the increase in hospital density. 

A possible explanation could be made for the grids with significantly negative effects. The infection was still considerably intensified near a few places with a low density of major hospitals. For instance, there are only two major hospitals situated at the north and south ends of the residential area between the Sha Lake and East Lake. Because of the geographical restriction of these natural elements, nearby residents have to rely on the only two existing hospitals, intensifying the risk of cross-infection during the COVID-19 epidemic. In addition, fallacy correlation between hospital and epidemic severity may exist for specific locations, implying the existence of other interfering factors. For instance, although there is not any major hospital near the Baibuting community which is situated at the northern end of Hankou, there was still a hotspot of confirmed cases. After investigation, we found it was likely a result of the large-scale public banquet for the Spring Festival celebration that might eventually incur COVID-19 spread, if some latent pathogen carriers participated in such an event.

### 3.5. The Influence of Commercial Facilities

**Figure 8:**
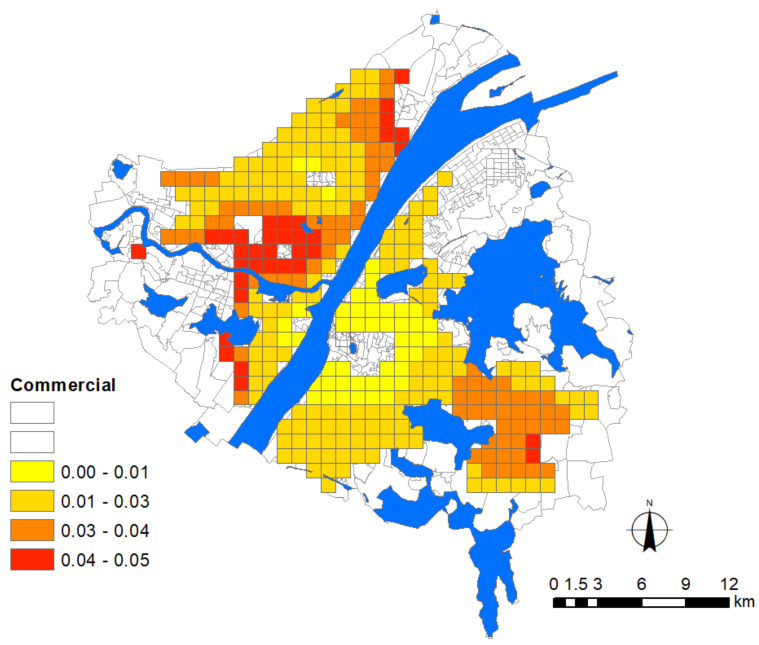
Areas significantly affected by the commercial facilities. The legend denotes the corresponding intensity of influence.

The result reveals that the epidemic situation is significantly affected by commercial density and such an influence is prevalent in the whole study area (Figure 8), particularly in most areas of Hankou and Hanyang, and the east–west commercial corridor from Wuchang District to Hongshan District, where large-scale commercial plazas and supermarkets are located. As the outbreak happened to occur just before the Spring Festival, the number of people who visited these commercial places to prepare for the festival purchase soared tremendously. Most of these commercial places are indoor spaces with relatively weak natural ventilation because doors and windows are mostly closed in order to keep warm. This situation further reduces the efficiency of air exchange from outside. Once the central air conditioning system is turned on, the circulating air will accelerate the spread of the pathogen and aggravate the epidemic situation in neighboring areas. 

For the urban fringe areas that are far away from the city centre, situations are more complex. For instance, the impact of commercial factors appears to be heavier for some fringe communities, such as Wangjiawan and Baibuting, where commercial supplies concentrate on some major supermarkets or malls, whereas the other fringe areas, such as Qingshan District and Gutian community, are subject to a non-significant impact with commercial density.

In contrast, the commercial factor around Ziyang Lake in the old city centre of Wuchang presents a non-significant effect on the epidemic situation. On the one hand, the large water coverage results in a low density of human settlement. Around this place, people’s movements and public activities mostly take place in the open air, in which the probability of virus spreading is much lower than that of closed indoor spaces. On the other hand, its surroundings are covered with some military and institutional compounds, and the strict control of the surrounding area covers not only commercial activities but also personnel movements. Therefore, the impact of commercial facilities on the disease was confined.

### 3.6. The Influence of Urban Growth

**Figure 9:**
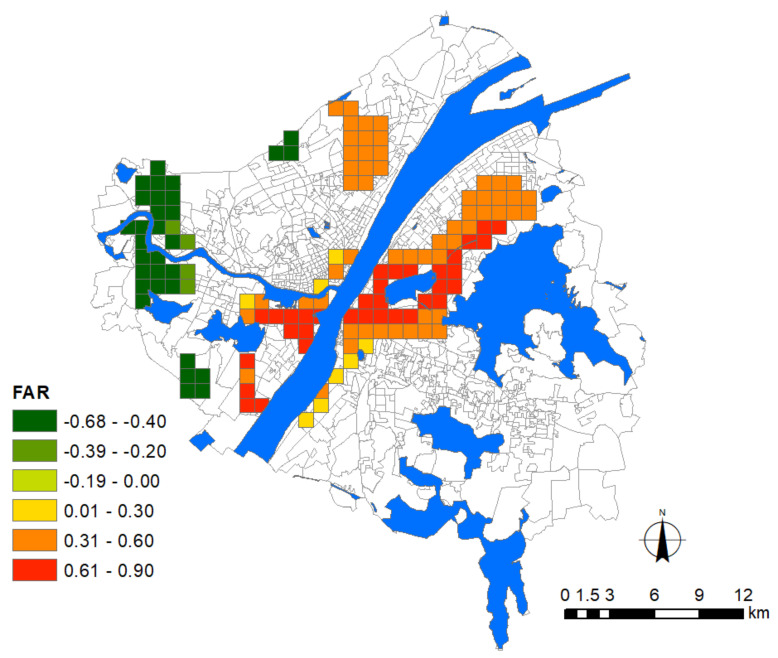
Areas significantly affected by the floor area ratio. The legend denotes the corresponding intensity of influence.

As a result, a continuous strip zone in which the epidemic situation is significantly affected by urban growth could be identified from the riverside area of Hanyang District to the broad area on the north-west bank of East Lake. The old downtown of Wuchang is also included in the affected area (Figure 9). Due to the existence of a large number of natural elements, many new real estate developments in the central area of Wuhan city are concentrated along this direction as the reserved land for residential construction is scarce and restricted. Many people prefer to live within such a decent living environment that is surrounded by waterfront landscapes, resulting in a higher residential occupancy than that of other urban fringe areas. In this circumstance, high-rise buildings were considered as an economical choice to meet the increasing accommodation needs, so as to save more in-between spaces to build the landscape, which is supposed to increase the value of the real estate. However, such a prototype forces residents to rely heavily on the elevator, which easily becomes a high-risk place due to human contact in such a narrowly confined space. Therefore, for these newly developed high-rise communities, the epidemic severity was intensified along with the increase in FAR.

In contrast, FAR exerted a significant negative effect on the epidemic situation for some communities on the upper stream of the Han River. Although some real estate developments have been finished in these urban fringe areas, the residential occupancy is still low, resulting in a fallacy where the epidemic seems suppressed when FAR increases.

### 3.7. The Influence of Aging

**Figure 10:**
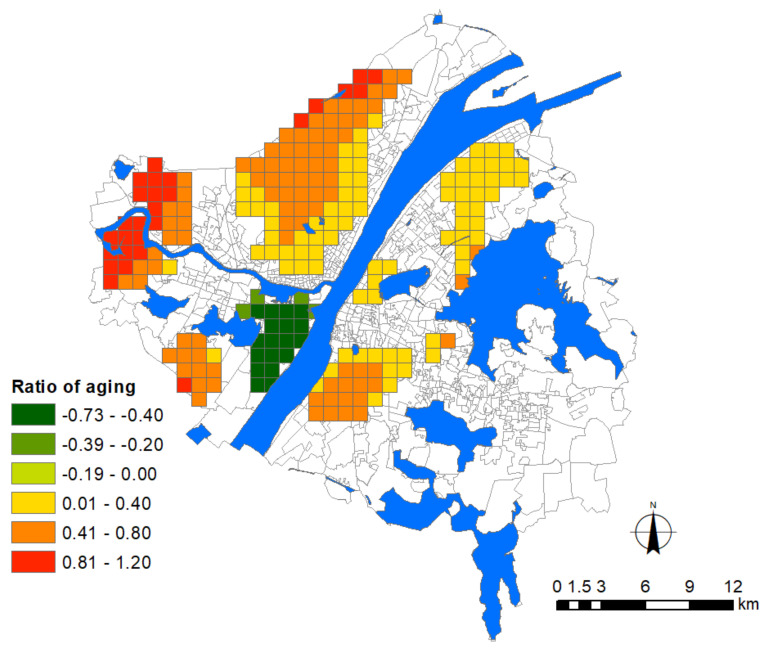
Areas significantly affected by the ration of aging population. The legend denotes the corresponding intensity of influence.

The result shows that the aging problem dominates most communities in Hankou and such a factor exacerbates the epidemic severity significantly. The impacted area overlaps with the epidemic distribution. Elderly people are reported as the most vulnerable population in the COVID-19 event [10], while Hankou is where aging communities are mostly concentrated in Wuhan city. Therefore, this area automatically became the epicenter that suffered the worst strike. Comparatively, the impact of aging on epidemic severity is low on the other side of the Yangtze River, and the impacted area with significance is much smaller and dispersed (Figure 10), mainly concentrating in Qingshan District to the north and the Baisha community to the south. For the broad area between the East Lake and the South Lake, where dozens of universities are located, the aging factor presents a non-significant effect on the epidemic severity. As university students account for the majority population in these communities, the epidemic situation was not seriously influenced by the aging factor.

Although the impact of aging is somewhat different for communities on both sides of the Yangtze River, its influence appears greater for communities of the urban fringe. The supply of urban support system is relatively scarce around these communities, particularly the medical resources. Therefore, residents who live away from urban centers are easily prone to panic when facing such a serious emergency, releasing more help-seeking data in these areas.

On the contrary, the epidemic severity turned out to be serious around the Zhongjiacun commercial loop in Hanyang District, although the aging ratio of this area is low. As was found, this place is very close to the high-risk epicenter, with two bridges directly connecting to the epidemic hotspot in Hankou. The frequent communication between the working class with better mobility might contribute to the epidemic diffusion.

### 3.8. The Influence of Subway

**Figure 11:**
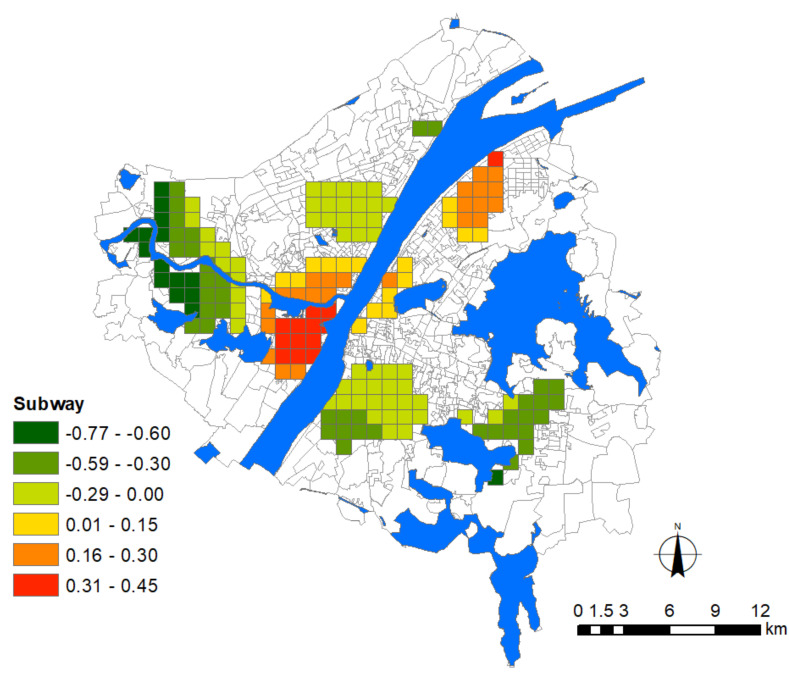
Areas significantly affected by the subway station. The legend denotes the corresponding intensity of influence.

At the adjacency place where two major rivers meet, the epidemic situation was intensified with increasing density of subway stations (Figure 11). This result further reinforces the hypothesis that the severity of the epidemic intensified although the aging ratio decreased around the Zhongjiacun commercial loop in Hanyang District. As a hub that connects Hankou and Wuchang, this place was under the influence on both sides. The younger population is subject to better mobility than seniors. In particular, for the working class, who heavily rely on subway for daily commuting, frequent contact via mass transportation exacerbated the epidemic severity in the area. A similar situation also took place near Jiyuqiao and Pangxiejia in Wuchang District, possibly because Line-2, which is the major artery line that connects both sides of the Yangtze River, sets up an important front station in this area before heading to the epidemic hotspot in Hankou. Another significantly impacted area appears in the residential area of the steel enterprise in Qingshan District, which is located near the terminal of Line-4, which a large number of residents live near; therefore the cumulative effect possibly exists to intensify the epidemic severity.

The newly built subway stations or those under construction are mainly distributed at some urban fringe areas, including Baisha and some communities on the upper stream of the Han River (Figure 10), with quite a low frequency of usage. Therefore, these areas present a significant inhibitory effect on the epidemic. The only exception is the Optics Valley Plaza, where a concentration of the epidemic emerged although the density of subway stations is very low. The situation is probably due to the commercial factors as discussed before, because it is adjacent to a very popular commercial district which is composed of several commercial streets across multiple blocks with the total length of 1.5 km. Featuring stylish architectural characteristics of European countries, this district is one of the most favorite places for shopping and leisure, playing a major role in the epidemic diffusion around this area.

### 3.9. The Influence of Land Use Mixture

**Figure 12:**
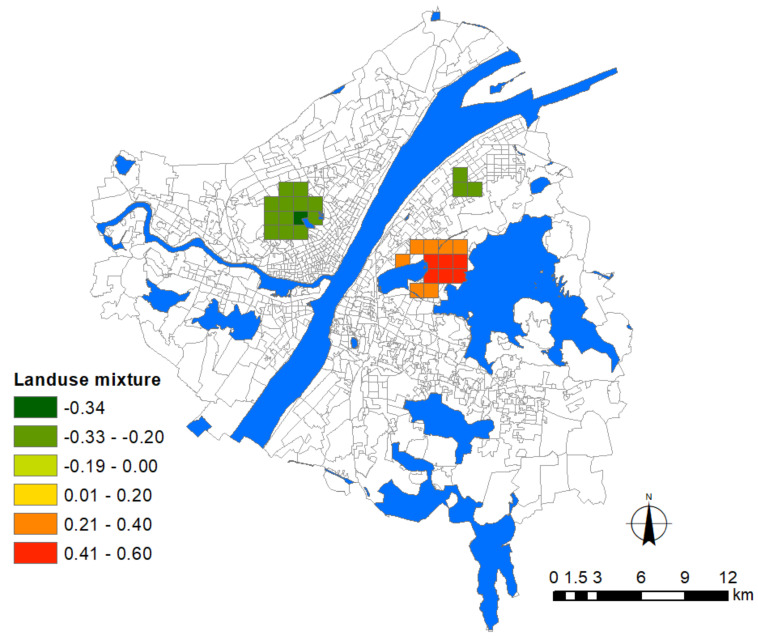
Areas significantly affected by the landuse mixture. The legend denotes the corresponding intensity of influence.

The result indicates that the impact of land-use mixture on the epidemic situation is relatively limited. Different effects appeared in Hankou and Wuchang (Figure 12). In Hankou, the significantly impacted area with negative effect exists among the in-between space of Northwest Lake, Zhongshan Park, and Fountain Park. This area generally belongs to the type of balanced community, in which people can enjoy spatial proximity to community services and the diversity of urban functions. According to Cervero, urban communities can be roughly categorized as three groups with different commuting patterns, i.e., the balanced community of residence and employment, the residence-based community, and the employment-based community [29]. In the balanced community, people can easily obtain basic needs within walking distance, as it reduces the frequency of long-distance commuting across different regions [30]. The frequency of traversing across surrounding high-risk areas could be avoided, particularly for the office staff who basically work within a designated area. In addition, some large-scale water pools that are dispersed in this area also play the role of barriers to reduce people’s mobility. 

In Wuchang, land-use mixture presents a significantly positive effect around the area between the Sha Lake and East Lake. As this area is mainly dominated by residence-based communities, residents’ commuting distance and their dependence on public transportation increases with a higher degree of land-use mixture [31], elucidating why mixed land-use to intensify the epidemic severity at this place.

## 4. Possible Future Strategies Based on the Reported Results

In the early stage of the outbreak, unexpected exposure to the contaminated environment in the hospital was prone to happen during medical treatments as the public had not formed a clear awareness of the characteristics of such a novel virus. After that, the infected or suspected population fluxed into major hospitals, not only leading to more serious cross-infection, but also accelerating the collapse of the local medical system. The well-developed commercial network and subway system facilitated the spread of this disease to a wider area within the city. 

Verification of these urban factors and their unevenly distributed effects will contribute to the development of specific planning and design responses for different areas in the city, making urban communities less susceptible when dealing with similar diseases in the future. According to the lessons and experience from this case, the construction of a multi-level emergency medical system should be fully considered and rationally configured in future urban planning, which could relieve the pressure of major hospitals and benefit precautions. For instance, the first level of local hospitals could serve for the diagnosis of suspected patients; the second level is the major hospitals, which are responsible for treating severe cases; the third level is the shelter hospitals, which serve to isolate patients with mild or moderate symptoms; while the fourth level consists of a large number of centralized quarantine facilities for receiving discharged patients. In addition, the distribution of emergency medical units, especially major hospitals, should be also planned with lower density and a certain distance in between. A high concentration of major hospitals as the single-level emergency medical system turns out to be less effective and even dangerous in dealing with infectious epidemics. Besides, there should be more caution in allocating large-scale indoor commercial facilities, in order to avoid the potential relay spread from hospitals to commercial facilities. Positive prevention strategies with detecting and sterilizing devices could be considered before customers enter these places.

In addition, high-density communities with high-rise buildings face higher risks of a severe impact of COVID-19. For some communities on the urban fringe, the aging problem exacerbated the situation and made them more vulnerable when encountering emergencies. More attention should be paid to establishing and improving the emergency medical system around these places. At present, the FAR of many residential quarters in Wuhan core area exceeds 2.0 or even reaches 3.0. Therefore, reasonable control of FAR for residential quarters, in particular around general hospitals, is necessary so as not only to create a livable environment, but also to ensure public health. In particular for those places located around major hospitals, as discussed before, a stricter control should be implemented. For those communities with high-rise residential buildings, each unit connects hundreds of residents who have to rely on elevators for circulation. Exploring preventive methods to improve the sanitary conditions of some frequently used public spaces, such as hallways, elevators, stairs, and handles, is also encouraged. 

Along with the rapid urbanization process, large cities heavily depend on rail transit, which may also serve as a fast passage of epidemic transmission. In particular for the “near subway housing” within one kilometer on both sides of the rail transit, the exposure risk is greater. Therefore, appropriate “buffering spaces”, such as street parks, open spaces, and green belts along the rail transit, should be considered and integrated into existing natural systems. In order to create a humanized urban environment, it is also helpful to encourage a variety of slow traffic modes within the 15-min life circle and to improve the pedestrian system and greenways around these nodes. In this way, the crowd of passengers who rely on the subway could be automatically reduced or dispersed via staggering peak travels, as is beneficial for the ease of disease prevention. Not only can these strategies relieve the pressure of public transportation, they can also play an important role in delaying the impact during emergent public health events.

## 5. Conclusions 

Wuhan encountered a serious attack in the first round of the COVID-19 pandemic which resulted in a public health social impact, including public mental health [32]. In this study, we adopted the Weibo data to infer the spatial pattern of the COVID-19 distribution of early stage in Wuhan city. The theoretical assumption that adopts some important urban factors that may influence epidemic severity was validated, such as urban growth, location of general hospital, commercial facilities, subway station, and aging ratio. The result further identified the unevenly distributed effects and spatial heterogeneity of these influencing factors in the city. More findings could be listed as follows. (1) The distribution and density of major hospitals exert a positive association with the epidemic situation. (2) The density of commercial facilities is the most prevalently distributed factor over the city that presents a positive association with the epidemic severity. (3) Newly developed residential quarters mainly distributed around the waterfront area of Hanyang and Wuchang with high-rise buildings face greater risks. (4) The influence of subway stations concentrates at the adjacency place where the three towns meet and near-terminal locations. (5) The global regression model shows that the influence of land-use mixture presents a significant association with the epidemic severity, whereas the local regression model shows that the influenced area is limited. (6) Last but not least, the community aging ratio dominantly affects Hankou to a broader extent than other areas in the city. To develop effective planning strategies in the future, a series of managerial implications that coordinate these urban factors have been proposed. For instance, the multi-level emergency medical system should be considered to replace the current single-level medical system which heavily relies on major hospitals, which should be distributed with lower density within a certain distance. Plans for allocating large-scale indoor commercial facilities should be more cautious to avoid the potential relay spread. Stricter control of FAR for residential quarters, in particular around general hospitals, is necessary. Besides this, slow traffic modes within the 15-min life circle should be encouraged by adding or improving the green belt, pedestrian system, and greenway around transit nodes. These spaces could be integrated into existing natural systems as an effective buffer-zone to play an important role in delaying the impact during emergent public health events. 

We end this study by acknowledging some limitations. First, as Weibo help data are sparse compared with the number of the confirmed cases, we adopted the kernel density to simulate the epidemic situation. In the future study, we will collect more data of confirmed cases with geo-information to ensure a more reliable result. Second, other urban factors that might have been neglected should be investigated to increase the model prediction. In addition, we should try to compare other models, e.g., spatial error and spatial lag, to optimize the model-fit. Once more data are expected to be available, the Bayesian conditional autoregressive model could be implemented to dive into this study with more advanced methods.

## Figures and Tables

**Table 1:** Statistics of the Weibo data and the confirmed cases.

Data	Jiang’an	Jianghan	Qiaokou	Hanyang	Wuchang	Qingshan	Hongshan	Total
Cumulative confirmed	6521	5137	6789	4661	7431	2773	4652	37,964
Weibo help	72	35	50	37	62	22	49	327
Chi-square	9.977							
Sig.	0.126							

**Table 2:** Summary of urban factors.

Categories	Factors	Description
Socio-demographic	Aging	The proportion of elderly people over 65 years old within the community.
Urban growth	FAR	The ratio is determined by dividing the gross floor area of buildings by the gross area of the lot on which buildings stand.
Urban facilities	Hospital density	The kernel density of measuring the probability of existence of the general hospital within a given area.
	Subway density	The kernel density of measuring the probability of existence of the subway station within a given area. The value is highest at the location of the given point and diminishes with increasing distance away from it.
	Commercial density	The kernel density of measuring the probability of existence of commercial facilities within a given area. The value is highest at the location of the given point and diminishes with increasing distance away from it.
	Road density	The ratio is determined by dividing the gross length of roads within a given grid by the gross area of the grid.
Land use	Land-use mixture	The land-use mixture measures the diversity of land use, such as the residential, commercial, industrial, etc. The Shannon-entropy is often calculated to denote the land-use mixture based on the proportion of land use types within the selected study area.

**Table 3:** OLS model results.

Variable	Estimates	Robust-S.E.	Robust-t	Robust-*p*	VIF	Model-Fit	
intercept	−0.002	0.034	−0.054	0.957		F	128.498
Aging (hundred/km^2^)	0.279	0.534	5.215	<0.001 ***	2.492	Wald	717.899
FAR	0.145	0.053	2.729	0.007 **	2.266	R2	0.646
Hospital density	0.333	0.121	2.754	0.006 **	2.530	Adjusted R2	0.641
Subway density	0.086	0.030	2.886	0.004 **	2.534	AICc	320.042
Commercial density	0.016	0.003	6.325	<0.001 ***	3.603		
Road density	0.002	0.004	0.528	0.598	1.515		
Land-use mixture	−0.075	0.038	−1.978	0.048 *	1.134		

Note: * *p* < 0.05; ** *p* < 0.01; *** *p* < 0.001.

**Table 4:** GWR model results.

Variable	Est. (Lower)	Est. (Upper)	S.E. (Lower)	S.E. (Upper)	Model-Fit	
intercept	−0.993	0.460	0.071	0.335	R2	0.871
Aging (hundred/km^2^)	−1.268	1.176	0.087	3.7146	Adjusted R2	0.822
FAR	−0.672	1.110	0.096	0.738	Local R2	0.263–0.855
Hospital density	−2.905	2.135	0.157	4.533	AICc	63.111
Subway density	−0.790	0.414	0.042	0.927		
Commercial density	−0.012	0.082	0.003	0.038		
Land-use mixture	−0.303	0.474	0.078	0.285		

**Table 5:** Spatial autocorrelation test.

Model	Moran’ I	z	*p*
Nulll	0.796	32.886	<0.01
OLS	0.611	25.252	<0.01
GWR	0.314	13.036	<0.01
